# Evaluation of anxiety symptoms and depression in the general albanian population during quarantine

**DOI:** 10.1192/j.eurpsy.2021.292

**Published:** 2021-08-13

**Authors:** F. Elezi, S. Tomori, G. Tafani

**Affiliations:** 1 Psychiatric Department, University Hospital Center, Tirana, Albania; 2 Pediatric Department, University Hospital Center “Mother Teresa”, Tirana, Albania; 3 Psychiatric Department, University Hospital Center “Mother Teresa”, Tirana, Albania

**Keywords:** covis-19, anxiety, depression, general population

## Abstract

**Introduction:**

During the COVID-19 pandemic, the Albanian authorities declared mandatory stay-at-home measures, closing businesses, schools and public places.

**Objectives:**

To investigate the impact of these immediate changes on the mental wellbeing of the population.

**Methods:**

Respondents (N=1678) from 18 to 60 years old were selected through a convenient sampling method. Questionnaires were administered online reporting time spent daily in the COVID-19 topic and genealities; the Patient Health Questionnaire-9 and Generalized Anxiety Disorder-7.

**Results:**

Findings suggest a significant negative correlation between age and anxiety scoring (r_(n=1678)_=-.121, p≤.001) and age and depression scoring (r_(n=1678)_=-.232, p≤.001), shown also on the ANOVA test for age and anxiety (F=6.019, p≤.05) and age and depression (F=20.326, p≤.05). Differences on the level of education resulted in a lower score of anxiety and depression respectively (F=3.524, p≤.05), (F=7.739, p≤.05) on respondents with higher education. Those who were jobless from the pandemic scored higher on anxiety and depression respectively (F=9.760, p≤.05) (M=6.21, ds=4.686) and (F=16.051, p≤.05) (M=8.18, ds=5.791). Significant differences were found related to different amounts of time spent on the COVID-19 topic, respectively for anxiety and depression (F=25.736, p≤.001), (F=5.936, p≤.003), with people who spend less than 1 hour scoring higher on depression (M=7.57, ds= 5.849) and those who spend more than 3 hours scoring higher on anxiety (M=6.76, ds=5.60).

**Conclusions:**

Higher education individuals, having a job and being in a romantic relationship relate to lower levels of depression during Covid-19 quarantine in Albania. Spending more time on the COVID-19 topic daily and being a female relate to higher level of anxiety.

**Disclosure:**

No significant relationships.

